# Strategies for ^1^H‐Detected Dynamic Nuclear Polarization Magic‐Angle Spinning NMR Spectroscopy

**DOI:** 10.1002/chem.202003463

**Published:** 2020-11-03

**Authors:** Maria Concistré, Subhradip Paul, Marina Carravetta, Ilya Kuprov, Philip T. F. Williamson

**Affiliations:** ^1^ Biological Sciences/Institute of Life Sciences University of Southampton Southampton SO171BJ UK; ^2^ School of Chemistry University of Southampton Southampton SO171BJ UK; ^3^ Sir Peter Mansfield Imaging Centre University of Nottingham Nottingham UK

**Keywords:** ^14^N NMR spectroscopy, amyloids, biomolecular NMR spectroscopy, dynamic nuclear polarization, solid-state NMR spectroscopy

## Abstract

Combining dynamic nuclear polarization with proton detection significantly enhances the sensitivity of magic‐angle spinning NMR spectroscopy. Herein, the feasibility of proton‐detected experiments with slow (10 kHz) magic angle spinning was demonstrated. The improvement in sensitivity permits the acquisition of indirectly detected ^14^N NMR spectra allowing biomolecular structures to be characterized without recourse to isotope labelling. This provides a new tool for the structural characterization of environmental and medical samples, in which isotope labelling is frequently intractable.

## Graphical abstract

**Dynamic nuclear polarization**: Proton‐detected ^1^H/^14^N dynamic nuclear polarization magic‐angle spinning NMR method provides insights into the structure of biomolecular systems without recourse to isotope labelling. The improvement in sensitivity permits the acquisition of indirectly detected ^14^N NMR spectra allowing biomolecular structures to be characterized without recourse to isotope labelling.

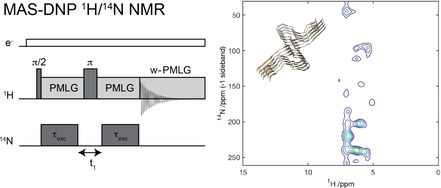

Although the characterisation of protein structure and function by magic‐angle spinning (MAS) solid‐state NMR is well established, sensitivity remains an issue: sample enrichment with the magnetically active ^13^C and ^15^N isotopes is normally necessary.^[1]^ Progress was recently made using either dynamic nuclear polarization (DNP) and fast (above 100 kHz^[2]^) MAS with proton detection.^[3]^ Because fast MAS and DNP are complementary, they could potentially be combined, but there are no reports of this to date—the engineering challenges involved in the probe design are formidable. However, promising homonuclear decoupling methods exist^[4]^ at MAS frequencies routinely accessible MAS‐DNP frequencies (<14 kHz).^[5]^ These techniques overcome the degradation in resolution caused by strong dipolar couplings^[6]^ under slow spinning, and in favourable cases have resulted in proton linewidths comparable to those measured in fully protonated proteins at fast (∼60 kHz) spinning.^[4b]^ Here we introduce an experiment that is a triple combination of DNP, slow MAS, and homonuclear decoupling, and use it to characterize amyloid fibrils composed of β_2_‐microglobulin—a fairly typical biological system where we found that the isotope enrichment was not required. We identified three key factors that made the implementation possible: a) attenuation of proton signals from the DNP matrix in a way that does not interfere with the DNP enhancement; b) elimination of the strong homonuclear dipolar interactions using homonuclear decoupling schemes; and c) transverse magnetisation management to minimise losses through paramagnetic relaxation and inhomogeneous broadening.

In ^13^C‐detected experiments on isotopically enriched samples, the signal from the DNP matrix is eliminated by using the different relaxation properties of the molecule under study and natural abundance ^13^C occurring in the DNP matrix.^[7]^ Our attempts to adapt this method for more abundant protons present in the sample and DNP matrix have not been successful.

We therefore opted for the removal of the protons from the buffer and DNP matrix washing the β_2_‐microglobulin fibrils repeatedly in deuterated buffer prior to lyophilization, an approach similar to that previous employed to study the surface chemistry of nanoparticles and MOFs.^[8]^ Furthermore, the DNP experiments were performed in a fully deuterated matrix with 10 mm AMUPol biradical in a glycerol‐d_8_/D_2_O (60:40 v/v) matrix. Despite previous studies highlighting the importance of retaining some protons within the DNP matrix to facilitate spin‐diffusion^[9]^ DNP enhancements (*ϵ*_on/off_) of 45 were obtained. These were comparable to the enhancements that were obtained for the protonated DNP matrix (glycerol‐d_8_/D_2_O, 60:30:10 v/v/v) (Figure 1) mirroring the observations of Kobayashi et al. who also reported similar levels of DNP enhancement in a fully deuterated matrix.^[8]^ Despite the effective removal of the water/glycerol signal this did lead to an 80 % reduction in the amide nitrogen signal in conventional ^15^N CP‐MAS spectra due to the loss of the solvent and exchangeable protons as a magnetization source (Figure S1). Although this sample preparation may compromise the application to some systems where H/D exchange occurs rapidly, for other biomolecular systems where exchange is slow (minutes/hours) such an approach is feasible as the low‐temperatures typically employed for MAS‐DNP studies quenches further exchange during the measurement.

**Figure 1:**
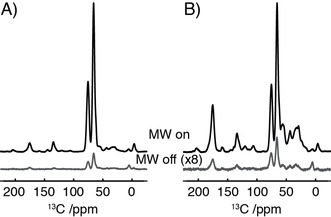
Comparison of DNP enhancement of the natural abundance ^13^C signal obtained for a 5 mg sample of ^15^N‐labeled‐β_2_‐microglobulin, in partially deuterated matrix (A), 10 mm AMUPol in glycerol‐d_8_/D_2_O/H_2_O (60:30:10)) and fully deuterated matrix (B), 10 mm AMUPol in glycerol‐d_8_/D_2_O (60:40)). Data recorded under MAS‐DNP at 14.1 T, 100 K and 9.8 kHz spinning frequency.

To ascertain the effect freezing and the presence the AMUPol biradical had on the proton lineshapes ^15^N detected HETCOR experiments were performed (Figure 2).

**Figure 2:**
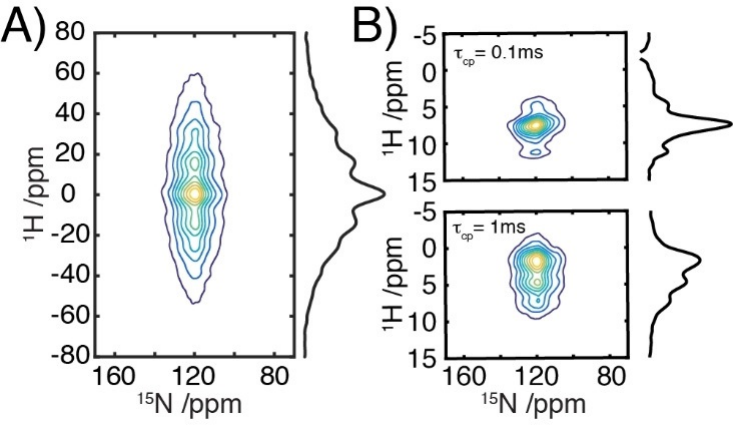
^1^H/^15^N HETCOR spectrum of ^15^N‐labeled‐β_2_m plus 10 mm AMUPoL in in glycerol‐d_8_/D_2_O (60:40); B) ^1^H/^15^N HETCOR spectra as in A but acquired using homonuclear decoupling during t_1_ for two different values of the mixing time *τ*_cp_=0.1 ms and *τ*_cp_=1 ms. Spectra have been recorded under DNP‐MAS at 14.1 T, 100 K and 9.8 kHz MAS rate.

In the absence of any homonuclear decoupling the proton spectrum was distributed into a family of sidebands spread over ∼80 kHz, as expected given the low spinning speed and the strong network of dipolar coupled protons at this temperature. Application of $wPML{G5}_{\bar{m}m}^{\bar{x}x}$ homonuclear decoupling^[4a]^ during the indirect dimension of the ^1^H/^15^N‐HETCOR experiment results in a significant improvement in resolution. Indeed, ^1^H T_2_′ spin‐echo measurements of the entire spectral envelope showed a 6–7‐fold enhancement in T_2_′. The improvement occurs despite the reduction in motional averaging of the dipolar couplings that accompanies the lower temperatures used when performing DNP. For shorter periods of cross‐polarization (100 μs, Figure 2 B, top) this results in an envelope of resonances spanning approximately 2–3 ppm, corresponding to the correlations between the ^15^N and the adjacent protons. For longer periods of cross polarization (1 ms, Figure 2 B, bottom) the strong network of proton homonuclear dipolar couplings leads to spin‐diffusion and a redistribution of the magnetization between all the sites within the protein, resulting in a 9 ppm envelope of resonances, whose intensities mirrors that typically observed for proteins.

The resolution observed in the homonuclear decoupled ^1^H/^15^N‐HETCOR data is not sufficient to resolve individual sites within the fibrils of the 13 kDa β_2_‐microglobulin. The distribution of chemical shifts in the amide region reflects that expected for the β_2_‐microglobulin, with the spectral envelope mirroring that expected based on the linewidths previously measured on microcrystalline protein preparations at ambient temperatures.^[4b]^ This observation indicates that under the conditions necessary for DNP, inhomogeneous and paramagnetic broadening are not limiting resolution as the linewidths observed are comparable to similar systems studies at ambient temperature in the absence of radical using homonuclear decoupling. These observations suggest that significant improvement may still be realized through the use of partial deuteration and/or faster spinning frequencies. Indeed, based on the studies of Mote & Madhu^[4b]^ even increases in spinning frequencies to 20–30 kHz, when combined with homonuclear decoupling may offer further improvements in resolution of ^1^H detected DNP measurements. The resolution attained does however indicates indirect proton detection represents an attractive route to the detection of nuclei with low gyromagnetic ratios.

Based on these studies, ^1^H/^14^N correlation experiments were performed on the fibrillar β_2_‐microglobulin. These experiments allow the use of unlabelled proteins, with enhanced resolution in the indirect dimension as the sites are resolved by both the isotropic ^14^N shift and the second order quadrupolar shift^[10]^ a property related to the size of the quadrupolar interaction that reflects the structures present within the protein. Unlabelled lyophilized β_2_‐microglobulin fibrils were resuspended in 10 mm AMUPol in a fully deuterated matrix (glycerol‐d_8_/D_2_O 60/40). To enhance proton resolution during detection a windowed PMLG (wPMLG) Scheme was applied (see the Supporting Information for a full description of the experiment performed). Typically, spectra of the amide region could be obtained in as little as 512 scans, allowing the acquisition of a full 2D ^1^H/^14^N correlation data set in as little as 14 h (Figure 3).

**Figure 3:**
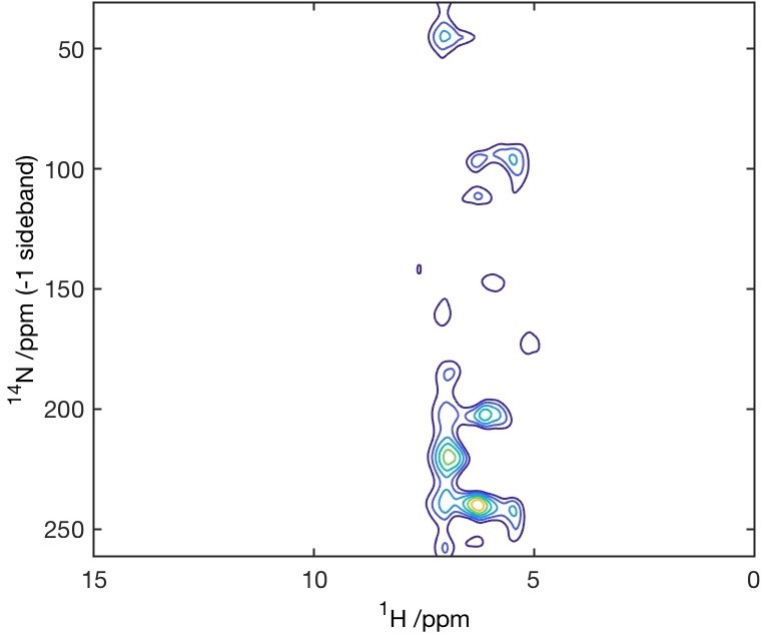
^1^H/^14^N correlation spectrum of unlabelled‐β_2_m‐protein fibrils doped with a 10 mm solution of AMUPol in glycerol‐d_8_/D_2_O (60:40). The experiment was performed under DNP‐MAS at 14.1 T, 100 K, 9.8 kHz spinning speed and using a PLMG homonuclear decoupling Scheme during the ^14^N pulses and ^14^N pulses evolution and wPMLG during proton acquisition.

As was predicted from the ^1^H/^15^N HETCOR data, the proton spectrum gives a family of resonances spread across 1–2 ppm in the amide region (at ∼7 ppm). The ^14^N resonances are spread across 50 ppm, with the most intense features centred between 200 and 250 ppm, with minor features as low as 50 ppm. The indirect dimension is rotor synchronised resulting in a 10 kHz spectral width with the resonances between 200 and 250 ppm representing the 1^st^ order sideband of the amide signal, resulting in a central resonance at 500 ppm. With the isotropic nitrogen chemical shift centred at 120 ppm (Figure 2), the $\delta_{Q}^{iso}$ can be calculated to be 380 ppm resulting in a C_Q_ of between 2.49 and 2.88 MHz (for details of calculations, see the Supporting Information).^[11]^ The large variation in $\delta_{Q}^{iso}$ ensures that the ^1^H/^14^N correlation spectrum provides exhibits greater resonance dispersion than the corresponding ^1^H/^15^N correlation spectrum which is resolved by the ^15^N isotropic chemical shift alone (For a direct comparison, see Figure S4). In addition to providing greater resonance dispersion, the $\delta_{Q}^{iso}$ of the amide nitrogen in the protein backbone is a valuable reporter on protein secondary structure, with significant variation between α‐helical and β‐sheet conformations.^[12]^ The observed C_Q_ of between 2.49 and 2.88 MHz for the amide nitrogen's is in the lower range of quadrupolar interactions observed in proteins. Such low C_Q_′s are typical of β‐stranded conformations such as that present in the cross‐β core of the β_2_‐microglobulin fibril.^[13]^ These results demonstrate the feasibility of conducting proton detected experiments using MAS‐DNP. By exploiting a proton free DNP matrix in conjunction with homonuclear decoupling sequences to supress the strong proton dipolar coupling network the resolution even at cryogenic temperatures (100 K) and in the presence of paramagnetic radicals is sufficient to assign chemical functionality even in complex biomolecular systems. Furthermore, despite the loses in resolution compared to studies at ambient temperatures with faster spinning frequencies, by exploiting techniques such as indirectly detected ^14^N NMR where improved resolution can be achieved by exploiting the $\delta_{Q}^{iso}$ , information regarding the structure of biomaterials can still be obtained. These findings clearly demonstrate that improved MAS‐DNP probes that can access faster spinning frequencies, direct proton detection may offer further enhancements despite the challenges of conducting studies in at cryogenic temperatures in the presence of paramagnetic agents. This highlights a potential role for using ^1^H detected DNP SS‐NMR to study biomaterials where labelling is either challenging or intractable.

## Conflict of interest

The authors declare no conflict of interest.

## Supporting information

As a service to our authors and readers, this journal provides supporting information supplied by the authors. Such materials are peer reviewed and may be re‐organized for online delivery, but are not copy‐edited or typeset. Technical support issues arising from supporting information (other than missing files) should be addressed to the authors.

Supplementary
