# Supplementary material for: Strategies for 1H‐Detected Dynamic Nuclear Polarization Magic‐Angle Spinning NMR Spectroscopy
Source: Chemistry. 2020 Nov 3;26(68):15852–4. doi: 10.1002/chem.202003463 (PMC7756879; doi:10.1002/chem.202003463)
Supplement: Supplementary file 1 — Supplementary [file CHEM-26-15852-s001.pdf]

# Chemistry–A European Journal

Supporting Information


## Strategies for $^{1}H$-Detected Dynamic Nuclear Polarization Magic-Angle Spinning NMR Spectroscopy


Maria Concistré,[b] Subhradip Paul,[c] Marina Carravetta,[b] Ilya Kuprov,[b] and Philip T. F. Williamson*[a]

## Author Contributions

M.C. Conceptualization: Supporting; Investigation: Lead; Methodology: Lead; Writing - Original Draft: Lead; Writing - Review & Editing: Lead
S.P. Investigation: Supporting; Methodology: Supporting; Writing - Original Draft: Supporting; Writing - Review & Editing: Supporting
M.C. Conceptualization: Supporting; Funding acquisition: Lead; Investigation: Supporting; Methodology: Supporting; Writing - Original Draft: Supporting; Writing - Review & Editing: Supporting
I.K. Conceptualization: Supporting; Funding acquisition: Lead; Investigation: Supporting; Methodology: Supporting; Project administration: Supporting; Writing - Review & Editing: Supporting
P.W. Conceptualization: Lead; Funding acquisition: Lead; Methodology: Lead; Project administration: Lead; Supervision: Lead; Writing - Original Draft: Lead; Writing - Review & Editing: Lead.

## Contents


1. Sample Preparation
2. Experimental Conditions and Pulse Sequences
3. $C_Q$ estimation
4. References

## 1. Sample Preparation

$\beta_2$-microglobulin was expressed and purified from inclusion bodies (IBs) in *E.coli*. Briefly, *E.coli* BL21 (DE3) were transformed with a pET11 a vector containing the coding sequence for the $\beta_2$-microglobulin gene. For unlabeled samples the *E.coli* were grown on Luria Broth (LB) (Sigma-Aldrich) supplemented with 100 $\mu$g/ml ampicillin at 37°C with 200 rpm shaking until an OD600 of 0.6-0.8 was reached. Expression was induced through the addition of IPTG to a final concentration of 1 mM and grown for a further 4 hours prior to harvesting by centrifugation at 3,000 rpm. For samples enriched in $^{15}$N the overnight culture was used to inoculate 500 mL of M9 media supplemented with 100 $\mu$g/ml ampicillin and grown to a OD600 of between 0.6 and 0.8. The bacteria were harvested by centrifugation at 4,000 x g and resuspended in 500 mL of minimal media containing 1 g $L^{-1}$ $^{15}$N ammonium chloride and 2 g $L^{-1}$ unlabeled glucose. After 8 hours the cells were harvested by centrifugation at 6900 x g for 20 minutes at 4°C and frozen until required for purification.

The bacterial pellet was resuspended in 30 mL HEPES (20 mM, pH= 7.4), and the cells broken by sonication using a stud sonicator. The IBs were resuspended in 25 mL of wash buffer (10 mM Tris-HCl pH 7.5, 2.5 mM $MgCl_2$, 0.5 mM $CaCl_2$) containing DNaseI and lysozyme and incubated for 1 hour before pelleting by centrifugation (20 min, 16000 x g). The IBs were washed a further 4 times by resuspending the them in Triton-buffer (50 mM Tris-HCl pH 8.0, 100 mM NaCl, 0.5% Triton X-100) and pelleting between washes (20 min, 16000 x g). Finally the IBs were dissolved in solubilization buffer (8M urea, 50 mM MES, 0.1 mM EDTA 0.2 mM DTT), incubating overnight at 4 °C. The solubilized material was then clarified by centrifugation (20 min, 16000 x g).

The solubilised $\beta_2$m was refolded by dilution at 4°C. The solubilised $\beta_2$m was first diluted 1:1 with refold buffer (100 mM Tris-HCl pH 8.0, 400 mM L-Arginine-HCl, 2 mM EDTA, 5 mM Glutathione-reduced, 0.5 mM Glutathione-oxidised, 0.1 mM PMSF), and then added drop wise at 0.1 ml/min using a peristaltic pump to ice cold refold buffer such that the final concentration of $\beta_2$m would be less than or equal to 5 $\mu$M. After refold, the $\beta_2$m was concentrated using a 5000 MWCO Kvick filtration system (GE Healthcare) to a concentration of ~2 mg/ml as determined by nanodrop.

Monomeric $\beta_2$m was isolated by size exclusion chromatography using a Sephadex 75 Hiload 16/60 column (GE Healthcare) equilibrated with gel filtration buffer (10 mM HEPES pH 7.4, 50 mM KCl, 0.1 % Sodium azide buffer). The column was run at a flow rate of 1 mL $min^{-1}$ with the sample loaded as a 2 mL aliquot. Fractions corresponding to monomeric $\beta_2$m were pooled and then concentrated to 1 mg/ml by centrifugal filtration. To form fibrils, $\beta_2$m was mixed 1:1 with a low pH sodium citrate buffer to lower its pH to 2.5. The solutions were then incubated at 37 °C with 200rpm orbital shaking for 3 days.

To facilitate proton detection, residual protonated buffer components were removed prior to resuspending the $\beta_2$m fibrils in a deuterated DNP matrix. The extent to which the protons were removed and its influence on the overall $^{15}$N spectra were monitored by $^1$H MAS-NMR and $^{15}$N CP-MAS studies. Washing of the samples four times with $D_2O$ significantly reduced the strength of the water resonance (Figure S1B). However to completely abolish the water signal the sample was lyophilized overnight resulting in spectra dominated by non-labile protons attached to the protein.

For DNP measurements, 5 mg of lyophilized β$_2$m fibrils were resuspended in a solution of 10 mM AMUPol (15-{[(7-oxyl-3,11-dioxa-7-azadispiro[5.1.5.3]hexadec-15-yl)carbamoyl][2-(2,5,8,11-tetraoxatridecan) in 60% glycerol-d$_8$, 40% $D_2O$. The sample was then packed into Bruker 3.2 mm sapphire rotor.

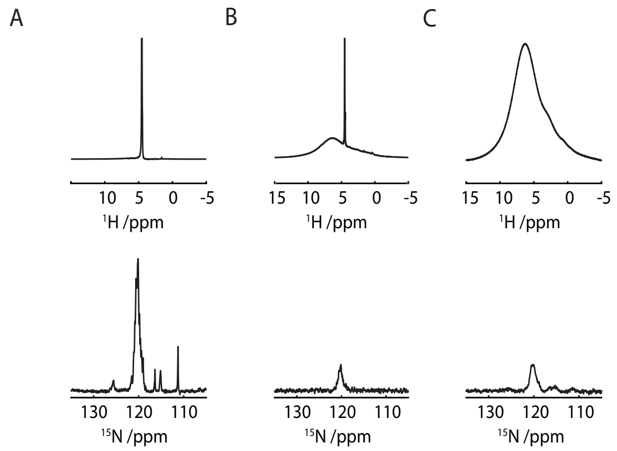


**Figure S1:** $^1$H spectra (upper row) and $^{15}$N CP spectra (lower row) of $^{15}$N labelled β$_2$m fibrils. (A) Before the washing; (B) after washing the sample 4 times in $D_2O$, (C) after lyophilization. Data acquired at 14.1T on a Varian DD2 spectrometer equipped with 1.6mm TR-MAS probe. Data acquired at 35 kHz, at 25°C. 15N CP-MAS spectra were acquired with a $^{15}$N spin lock field of 87.5 kHz adiabatic sweep[1] of the $^1$H rf amplitude optimised to match the n=+1 Hartmann-Hahn Condition. During acquisition protons were decoupled using 120 kHz SPINAL decoupling[2] .

## 2. NMR Experiments.

All DNP experiments were performed on a Bruker Avance III spectrometer operating at 14.1 T (600 MHz of $^{1}H$ Larmor frequency) equipped with a gyrotron oscillator at 395 GHz and a low temperature, triple-resonance 3.2 mm MAS probe tuned to $^{1}H/^{13}C/^{14}N$ or $^{1}H/^{13}C/^{15}N$. Spectra were recorded at a temperature of 100 K with a MAS frequency of 9.8 kHz. Data were processed in matNMR [3].

Carbon-13 MAS spectra were acquired with cross-polarization from protons to $^{13}C$ using a linear ramp from 90-100% proton amplitude and a 50 kHz $^{13}C$ spin-lock field. Optimal transfer was obtained at the n=+1 Hartmann-Hahn condition with a 1.75 ms contact time. During acquisition protons were decoupled with 100 kHz $SW_f$-TPPM[4] decoupling with a 5.1 μs pulse and ± 15° phase flip.

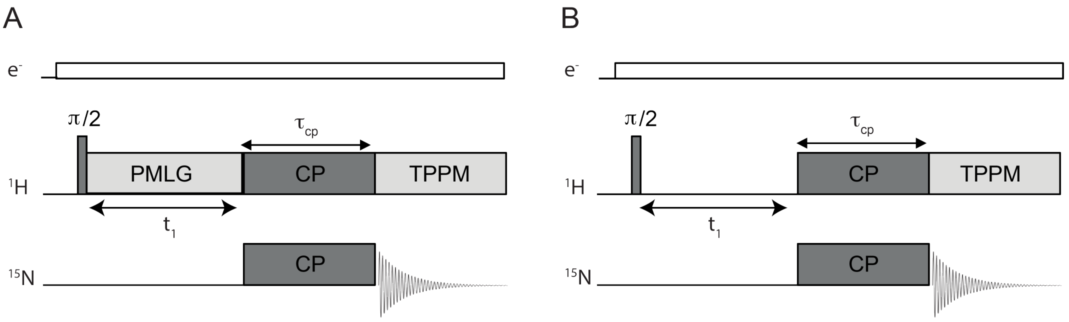


**Figure S2.** Pulse sequence used for the $^{1}H/^{15}N$ HETCOR correlation spectra with (A) and without (B) PMLG homonuclear decoupling during acquisition and $t_1$

$^{1}H/^{15}N$ correlation spectra were acquired using a standard hector correlation sequence (Figure S2). Ramped cross-polarization from $^{1}H$ to $^{15}N$ was achieved. The $^{15}N$ spin-lock field was set to 73 kHz and the $^{1}H$ rf amplitude matched to the n=+1 Hartmann-Hahn condition. Unless stated the contact pulses were of 1ms duration. To enhance the proton resolution in the indirect dimension 15 PMLG cycles of homonuclear decoupling were applied. All 2D spectra were recorded with States-TPPI[5] with 512 scans for each of the 64 increments with a 5 second recycle delay. Spectra were zero filled to 2048 points in each dimension and an exponential linebroadening of 50 Hz applied prior to Fourier Transform.

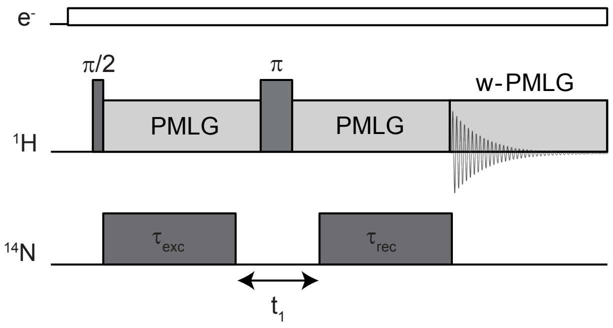


**Figure S3.** Pulse sequence for indirect detection on $^{14}N$ via $^{1}H$. The indirect period ($t_1$) must be multiple of the rotor period, and excitation and reconversion pulse widths must be chosen such that the spin echo period is rotor synchronised.

$^{1}H/^{14}N$ 2D correlation spectra were recorded with the pulse sequence shown in Figure S3. The excitation and reconversion pulses applied to $^{14}N$ were rotor synchronized  and at an RF amplitude of 30 kHz. Two-dimensional data were acquired using States-TPPI[5]. Unless stated data were acquired with a 5 second recycle, with 32 rotor synchronized $t_1$ increments. PMLG decoupling was applied during $^{14}N$ excitation, evolution and reconversion, with similar windowed PMLG applied during observation. Typically 15 PMLG cycles were applied per rotor period. Spectra were zerofilled to 2048 points in either dimension, and exponential linebroadening of 50 Hz was applied in the F1 dimension before 2D Fourier transform.

## 3. $C_Q$ estimation

The $^{14}$N second order isotropic quadrupolar shift (SOIQS) is given by[6]:

$$^{14}N\ \delta_Q^{iso} = \frac{3}{40}\left(\frac{\chi_Q}{\nu_0}\right)^2 10^6 \qquad [1]$$

where $\nu_0$ is the Larmor frequency and $\omega_Q$ is the quadrupolar product:

$$\chi_Q = C_Q\sqrt{1+\frac{\eta^2}{3}} \qquad [2]$$

where $C_Q$ is the quadrupolar coupling constant and $\eta$ the asymmetry parameter.

By using equantion [1] and [2] we can estimate the distribution of quadrupolar couplings present.

Subtracting from the centre of the amide region of the $^{14}$N spectrum of $\beta_2$m, 450 ppm, the centre of the amide region of the $^{15}$N spectrum, 120 ppm, one can determine an ‘‘average’’ amide SOIQS in $\beta_2$m of 380 ppm at 14.1 T. From eqn [1] and [2], one can determine this to be consistent with $C_Q$ values of 2.49–2.88 MHz, without any knowledge of the asymmetry parameter.

## 4. Comparison of resolution in $^{14}N/^{1}H$ and $^{15}N/^{1}H$ correlation spectra.

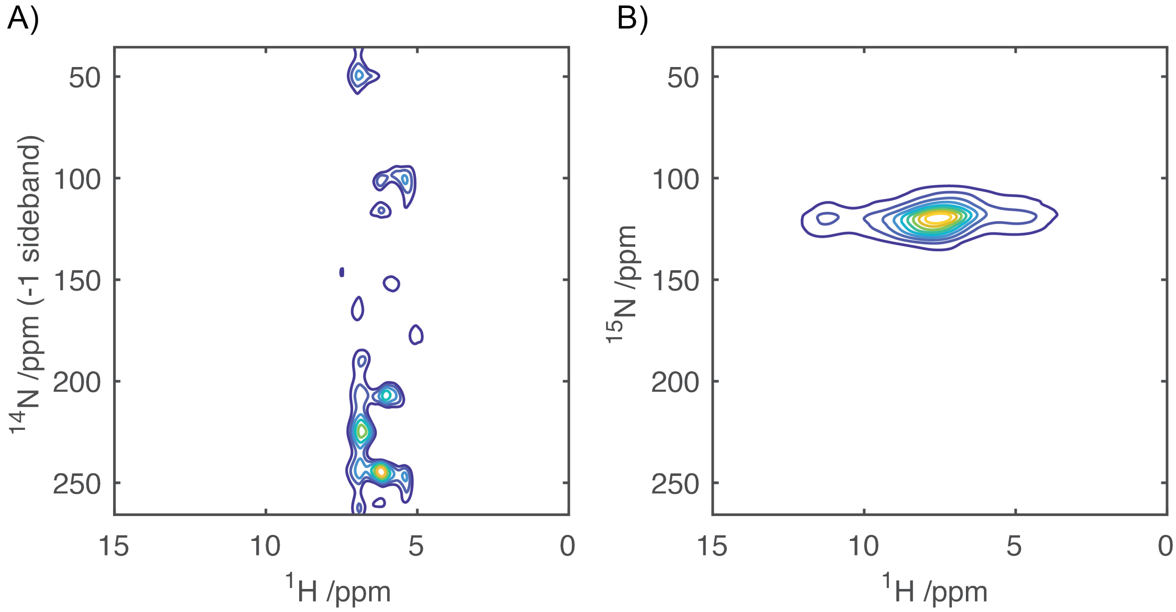


**Figure S4.** Comparison of the resolution in the $^{1}H$ detected $^{14}N$ resolved spectrum (A) and the $^{15}N$ detected $^{1}H$ resolved 2D correlation spectra (B). Within the $^{1}H/^{14}N$ correlation spectra resonances are resolved on the basis of the sum of their isotropic chemical shift and the second order isotropic quadrupolar shift ($\boldsymbol{\delta_Q^{iso}}$). The $\boldsymbol{\delta_Q^{iso}}$ is dependent upon the size of the quadrupolar interaction as described above, a parameter that can vary by 100's of kHz even for nitrogen in exhibiting subtle differences in hydrogen bonding such as those observed between amide nitrogens in protein backbones adopting $\alpha$-helical as opposed to $\beta$-sheeted conformations. In addition to providing valuable structural information, this also provides addition resolution in the 14N dimension. In contrast, the resolution in the 15N dimension of a $^{1}H/^{15}N$ correlation spectrum is determined by the isotropic chemical shift which in proteins shows relatively small dispersion (~10-15 ppm as opposed to the 100's ppm observed in the $^{14}N$ spectrum). Both spectra acquired under the conditions described in the Materials and Methods.

## References


1. Hediger, S. et al. *Chem. Phys. Lett.* **240**, 449-456 (1995).
2. Fung, B.M. et al. *J. Magn. Res.* **142**, 97-101 (2000).
3. Van Beek, J.D. *J. Magn. Res.* **187**, 19-26 (2007).
4. Chandran, C.V. et al. *Magn Reson Chem* **46**, 943-947 (2008).
5. Marion, D. et al. *Journal of magnetic resonance* **85**, 393-399 (1989).
6. Samoson, A. *Chem. Phys. Lett.* **119**, 29-32 (1985).
